# Clinical Assessment of an Ipsilateral Cervical Spinal Nerve Block for Prosthetic Laryngoplasty in Anesthetized Horses

**DOI:** 10.3389/fvets.2020.00284

**Published:** 2020-06-02

**Authors:** Tate B. Morris, Jonathan M. Lumsden, Colin I. Dunlop, Victoria Locke, Sophia Sommerauer, Samuel D. A. Hurcombe

**Affiliations:** ^1^Department of Clinical Studies, New Bolton Center, University of Pennsylvania, Kennett Square, PA, United States; ^2^Randwick Equine Centre, Randwick, NSW, Australia; ^3^Department for Companion Animals and Horses, Equine Clinic, University of Veterinary Medicine Vienna, Vienna, Austria

**Keywords:** locoregional, anesthesia, laryngoplasty, hemiplegia, ultrasound-guided, nociceptive, nerve, horse

## Abstract

The nociceptive blockade of locoregional anesthesia prior to surgical stimulation can decrease anesthetic agent requirement and thereby potential dose-dependent side effects. The use of an ipsilateral second and third cervical spinal nerve locoregional anesthetic block for prosthetic laryngoplasty in the anesthetized horses has yet to be described. Anesthetic records of 20 horses receiving locoregional anesthesia prior to laryngoplasty were reviewed and compared to 20 horses of a similar patient cohort not receiving locoregional anesthesia. Non-blocked horses were 11 times more likely to require adjunct anesthetic treatment during surgical stimulation (*P* = 0.03) and were 7.4 times more likely to receive partial intravenous anesthesia in addition to inhalant anesthesia (*P* = 0.01). No horse in the blocked group received additional sedation/analgesia compared to the majority of non-blocked horses (75%) based on the anesthetist's perception of anesthetic quality and early recovery movement. No difference in recovery quality was observed between groups (*P* > 0.99). Cervical spinal nerve locoregional anesthesia appears well-tolerated and useful in reducing cumulative anesthetic agent requirement and may decrease the need for additional sedation/analgesia in horses undergoing anesthetized prosthetic laryngoplasty.

## Introduction

According to a significant multicenter inquiry, more than 58% of perioperative fatalities in “non-colic” horses are due to either perianesthetic cardiovascular failure or musculoskeletal injury sustained during anesthetic recovery (1). Therefore, methods to optimize an uneventful and stable anesthetic period followed by good-quality recovery are critical. Modification of the anesthetic or sedative type and amount used is one such way, because all may promote or enhance anesthesia, but they are not without potential deleterious effects. The volatile anesthetics isoflurane and halothane have been shown to produce dose-dependent cardiopulmonary depression (2), whereas intravenous anesthetic drugs such as the *N*-methyl-d-aspartate-receptor antagonist ketamine can result in detrimental excitation or ataxia during recovery (3). Commonly used α2-adrenergic receptor agonists produce dose-dependent cardiovascular depression, decreased gastrointestinal motility, and ataxia (4), and opioids can have central nervous system stimulatory effects, as well as decrease gastrointestinal motility (4). Common theory states that, by combining multiple agents at smaller doses, adverse dose-dependent effects may be mitigated.

Local anesthetic agents can reduce other anesthetic agent requirement by inhibiting the transmission of action potentials of myelinated Aδ and unmyelinated C fibers nociceptive input via voltage-gated Na^+^ channel blockade (5–8). Despite routine use, controlled studies evaluating the effects of locoregional anesthetic nociceptive blockade in equine surgical patients are scarce. It has, however, been demonstrated that intratesticular infiltration with 2% lidocaine prevented a significant increase in mean arterial pressure (MAP) during castration of isoflurane anesthetized horses (9). Similarly, locoregional infiltration of 2% lidocaine decreases the likelihood of patient movement during total intravenous anesthesia, as well as the frequency of incremental adjunct anesthetic dosing (10). Both studies highlight the benefit of locoregional anesthesia in an anesthetic protocol.

In 2018, Campoy et al. described an ultrasound-guided locoregional anesthetic cervical spinal nerve block for use in horses through blockade of the ipsilateral ventral branch of the second cervical spinal nerve (C2) combined with subcutaneous perineural infiltration of the ipsilateral third cervical spinal nerve (C3) cutaneous branches (hence forth referred to as C2–C3) during sedated, standing prosthetic laryngoplasty (11). A conscious, sedated horse will respond to surgical stimuli in the absence of nociceptive blockade; therefore, locoregional anesthesia is essential. The unconscious, anesthetized horse responds to surgical stimuli in accordance to their depth and plane of anesthesia. This response can be controlled by deepening anesthesia with an increase of volatile anesthetic delivered and/or additional intravenous anesthetic agents; thus, nociceptive blockade is non-essential. However, this methodology increases agent requirement and potential dose-dependent side effects. There is clinical recognition that horses requiring supplementary intravenous anesthetic agent to prevent or control limb movement while under inhalation general anesthesia are more likely to undergo poor-quality recovery, despite there being no definitive scientific literature to confirm this clinical observation. Currently, horses undergoing anesthetized prosthetic laryngoplasty are managed in this manner.

With a reported perianesthetic crude procedural mortality rate of 7.0 deaths per 1,000, higher than either exploratory celiotomy (6.7) or fracture repair (1.9), anesthetized prosthetic laryngoplasty is not a totally benign procedure (12) Some hold a comparative clinical impression that these horses demonstrate inferior anesthetic recovery quality, which could contribute to perianesthetic mortality. Although unsubstantiated in the scientific literature, this impression is influenced by observations of paddling while laterally recumbent in early recovery, a lack of or decreased sternal phase, earlier and unsuccessful attempts to stand, incoordination or ataxia after rising, weakness, and return to recumbency requiring multiple attempts to stand. While not elucidated and likely multifactorial, explanations for such could include the perception of altered airway mechanics, postoperative pain, an increased amount of anesthetic used, and/or the administration of additional ataxia-potentiating analgesia or sedation during recovery.

Therefore, the use of locoregional anesthesia to provide nociceptive blockade in anesthetized horses undergoing laryngoplasty may improve anesthetic quality through lower anesthetic agent requirements and promote smoother recoveries.

The aim of this clinical report is to describe and investigate the effect an ipsilateral local anesthetic cervical spinal nerve block has on anesthetic parameters, anesthetic requirements, and recovery in anesthetized horses undergoing prosthetic laryngoplasty in a specialty practice clinical setting. We hypothesized that horses receiving a C2–C3 block would have a decreased cumulative anesthetic agent requirement and exhibit equal or improved recovery quality compared to horses not receiving the block.

## Materials and Methods

### Study Design

This was a retrospective, clinical case-control study. The anesthetic records of horses that underwent anesthetized left-sided prosthetic laryngoplasty at Randwick Equine Centre (R.E.C.), Sydney, Australia, from June 2016 to March 2017 were examined. All cases had surgery performed by the same surgeon in both blocked and non-blocked groups.

Horses included in the blocked group had C2–C3 performed via ultrasound guidance following induction of general anesthesia using a total of 10 mL of 0.5% bupivacaine HCl (bupivacaine injection; Pfizer Australia, Sydney, New South Wales, Australia) by a single veterinary anesthetist. These cases were enrolled from December 2016 through March 2017.

Horses in the non-blocked group had laryngoplasty performed from June 2016 to November 2016 and were enrolled in reverse chronological order until equal numbers of blocked and non-blocked horses with complete anesthetic records were available. Records with missing data points were excluded from assessment.

### Procedure(s)

#### Perioperative Treatment

All horses were classified prior to anesthesia using the American Society of Anesthesiologists (ASA) physical status classification scheme (I–V, including an E to denote any emergent case no matter class). All horses received procaine penicillin G [22,000 IU/kg intramuscularly (IM), ilium Propercillin; Troy Laboratories Pty. Ltd., Glendenning, New South Wales, Australia], gentamicin sulfate [6.6 mg/kg intravenously (IV), ilium Gentam 100; Troy Laboratories Pty. Ltd.], phenylbutazone (4.4 mg/kg IV, Salbute; CEVA Animal Health Pty. Ltd., Glenorie, New South Wales, Australia), dexamethasone (0.5 mg/kg IV, ilium Dexapent; Troy Laboratories Pty. Ltd.), and tetanus toxoid vaccine (1 mL IM, Equivac® T Vaccine; Zoetis Australia Pty., Rhodes, New South Wales, Australia). Horses were premedicated for general anesthesia using acepromazine (0.01–0.03 mg/kg IV, A.C.P. 10; CEVA Animal Health Pty. Ltd.), xylazine (up to 1 mg/kg IV/IM, Thiazine 100 Injection; CEVA Animal Health Pty. Ltd.), and methadone (0.1 mg/kg IM, ilium Methadone Injection; Troy Laboratories Pty. Ltd.).

#### Anesthetic Protocol

Horses were then anesthetized using a guaifenesin (40–90 mg/kg IV, ilium Guaiphenesin Injection; Troy Laboratories Pty. Ltd.) made to 5% solution using glucose (glucose 5% intravenous infusion BP; Baxter Healthcare Ltd., Old Toongabbie, New South Wales, Australia) followed by thiopental IV (5.6–6.0 mg/kg IV, Thiobarb Power; Jurox Pty. Ltd., Rutherford, New South Wales, Australia). All horses were orotracheally intubated, mechanically hoisted, and positioned on the surgical table in right lateral recumbency. Horses were instrumented with a three-lead electrocardiograph, pulse oximeter, capnograph, and an arterial catheter placed. Heart rate and rhythm, percent oxygen saturation, end-tidal CO_2_, and direct arterial blood pressure were routinely monitored. A semiclosed, circle rebreathing circuit was used to deliver anesthetic gas mixture (AAS Equine Trolley Machine; Advanced Anaesthesia Specialists, Gladesville, New South Wales, Australia). Anesthesia was maintained using either halothane (Halothane BP; Pharmachem, Eagle Farm, Queensland, Australia) or isoflurane (ISOTHESIA™; Henry Schein Inc., Melville, NY, USA) in 100% oxygen. Horses were allowed to breathe spontaneously or placed on intermittent positive pressure ventilation using a compatible stand-alone pressure cycled ventilator (LAV-3000, JD Medical Distributing Co., Phoenix, AZ, USA) if spontaneous ventilation fell below 5 breaths per minute or if Paco_2_ was >70 mm Hg. When mechanically ventilated, peak inspiratory airway pressure was maintained between 25 and 30 cm H_2_O. Additional boluses of anesthetic agents to deepen the plane of anesthesia were administered at the anesthetists' discretion based on several markers of anesthetic depth including spontaneous movement, assessment of muscle tone, lacrimation, nystagmus, and maintenance of a modified Guedel's anesthetic depth stage 3, plane 3 (central pupil position, medium pupil size, depressed palpebral reflex, depressed corneal reflex, minimally to moderately depressed heart rate/respiratory rate/blood pressure) throughout the procedure. For this study, an additional anesthetics was any agent administered or agent increased to deepen the plane of anesthesia and include an increase in percent inhaled volatile anesthetic and administration of a partial intravenous anesthetic solution (1 L 5% guaifenesin, 2000 mg ketamine, 1,000 mg xylazine) titrated to effect or a thiopental or ketamine (ilium Ketamil Injection; Troy Laboratories Pty. Ltd.) bolus. Dobutamine infusion (up to 2 μg/kg/min IV, Dobutamine-Hameln; Siegfried Hameln GmbH, Hameln, Germany) was administered and titrated to effect to maintain MAP >60 mm Hg.

#### C2–C3 Block Technique

**Figure 1:**
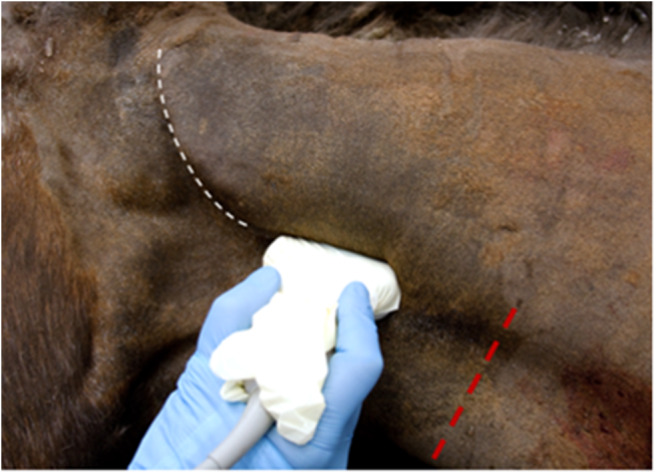
Correct ultrasound orientation, centering the probe ~6 cm caudal to the wing of the atlas (white line) and ventral to the palpable tendon of the *longissimus atlantis* m. Red line indicates the site of subcutaneous infiltration caudal to the surgical site.

**Figure 2:**
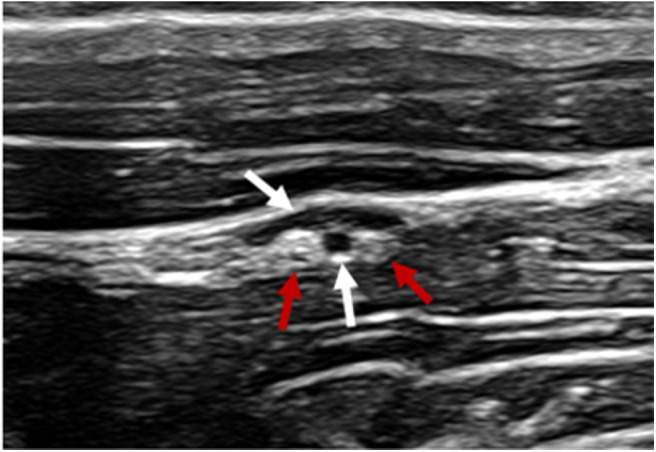
Ultrasonographic image of the c2-associated neurovascular bundle within the fascial plane created by the surface of the *cleidomastoideus* m (near field), *longissimus atlantis* m. (far field) and tendon of the *longissimus atlantis* m. Vasculature denoted by white arrows. Ovoid structures of mixed echogenicity consistent with nervous tissue denoted by red arrows.

**Figure 3:**
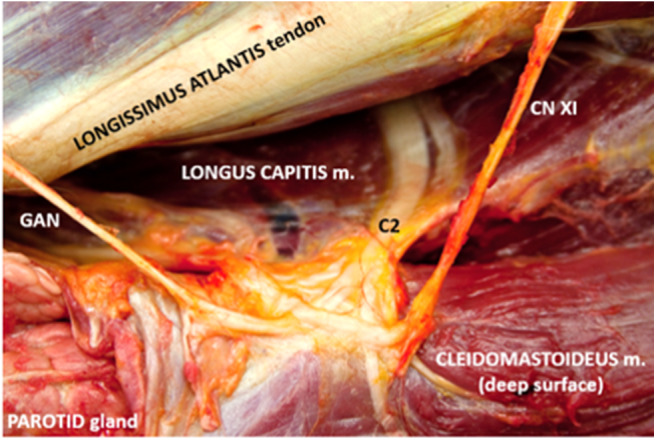
Corresponding anatomic dissection of c2-associated neurovascular bundle and site of local anesthetic deposition. Note several branches of c2 including the origin of the great auricular nerve (GAN) in addition to the dorsal branch of the spinal accessory nerve (CN XI).

Following clipping and aseptic preparation of the surgical site, the neurovascular bundle associated with the ipsilateral second cervical spinal nerve (C2) was identified sonographically using a technique similar to that previously described (11). Briefly, using a 12-MHz linear array transducer with compatible portable ultrasound machine (MyLab Alpha model 7400; Esaote S.p.A., Genova, Italy) positioned just ventral to the wing of the atlas at the level of parotid tissue, the operator scanned caudally with the probe oriented axially with a slightly dorsal angle (Figure 1) to find the fascial plane between the cleidomastoideus and longus capitis muscles. Continuing caudally while ventral to the tendon of the longissimus atlantis muscle, the C2-associated neurovascular bundle was identified within the fascial plane (Figures 2, 3) ~6 cm caudal to the wing of the atlas.

A 90-mm, 18-gauge spinal needle (UNIEVER Disposable Spinal Anesthesia Needle; UNISIS Corp., Saitama, Japan) was advanced in-plane with the ultrasound transducer to the caudal aspect of the neurovascular bundle and 0.5% bupivacaine instilled. Hydrodissection was noted. Subsequently, additional 0.5% bupivacaine was injected subcutaneously ~10 cm caudal to the proposed surgical incision as described previously (Figure 1). A total of 10 mL of 0.5% bupivacaine was divided between the two sites.

#### Surgery

A left-sided prosthetic laryngoplasty was then performed using a standard approach including dissection between the thyro-/cricopharyngeus muscles and the passage of two 7-metric coated braided polyester sutures (Ti-Cron™, COVIDIEN™; Medtronic, Minneapolis, MN, USA) within the caudal cricoid cartilage and left arytenoid muscular process. Prior to suture tying, arytenoid abduction was assessed by laryngeal video-endoscopic examination. Following lavage with sterile 0.9% NaCl (sodium chloride 0.9% 1,000 mL; Fresenius Kabi Australia Pty. Ltd., Mount Kuringai, New South Wales, Australia) infused with 1 g of ceftiofur sodium (Accent®; Zamira Life Sciences Pty. Ltd., Kenmore, Queensland, Australia), reapposition of the thryo-/cricopharnygeus muscles and a two-layered subcutaneous suture closure were performed (2-0 Vicryl®, Ethicon®; Johnson & Johnson, Bridgewater, NJ, USA). The skin was apposed with stainless-steel skin staples (Henry Schein® Skin Stapler; Henry Schein Inc.) and the site covered with a hypoallergenic polyacrylate adhesive tape (Fixomull® Stretch; BSN Medical GmbH, Hamburg, Germany). All horses had the same laryngoplasty technique performed, and no horses were undergoing a repeat laryngoplasty procedure. Postoperatively, all horses received a left-sided laser ventriculocordectomy in combination with a right-sided ventriculectomy and vocal cord transection performed standing under light sedation using a diode laser.

#### Recovery

All horses were recovered with the same recovery environment (i.e., the same recovery stall). Prior to arrival in the recovery stall, regardless of treatment group, horses received 20 mg methadone and 5 mg acepromazine IM at the anesthetist's discretion after evaluation of anesthetic quality including anticipation of a poor recovery based on the patient's response to surgical stimulation, administration of adjunct anesthetic, and a historical perspective of postlaryngoplasty general anesthetic recoveries. Moreover, intravenous xylazine was administered in the event of early spontaneous movement or rapid nystagmus either *en route* or once in the recovery stall. If apneic, respiratory support was provided via 100% oxygen demand valve (JDM-5040 Equine Demand Valve; JD Medical Distributing Co.) at a rate of 1 to 2 breaths per minute until spontaneous respiration. Once horses were spontaneously breathing, all horses were left to free-recover within the enclosed recovery stall. The cuff remained inflated, and orotracheal tubes were diverted out of the mouth at the level of the mandibular diastema, secured around the base of the ear with white adhesive tape and left indwelling for recovery. Oxygen insufflation was maintained throughout recovery at a rate of 10 L/min. Horses were monitored via video surveillance and extubation performed upon standing. Commonly used in clinical practice but unvalidated trichotomous descriptive subjective scale (good, fair, or poor) was used to rate the quality of each horse's recovery based on a combination of the following criteria: length of recovery time and number of attempts to stand and behavior within the recovery box (i.e., nystagmus, paddling, uncoordinated movements, weakness). Recovery grade was recorded on the anesthetic record by the anesthetist. Unfortunately, recovery video surveillance was not recorded for independent review.

### Statistical Analysis

All data were entered into a spreadsheet. Data were analyzed using commercial software (Microsoft Excel, Redmond, WA, USA; GraphPad Prism v8, San Diego, CA, USA).

Continuous data were assessed for normality using the Kolmogorov–Smirnov test. Data are expressed as mean ± standard deviation for normally distributed data and median (range) for non-normally distributed data. Differences between groups were assessed using either a *t*-test or Mann–Whitney *U*-test depending on the data normality. Categorical data are expressed as proportions and percentages and were assessed using Fisher exact testing with odds ratios and 95% confidence intervals calculated. Effect of volatile agent administered and block status in the patient on anesthetic recovery time was evaluated using a mixed-effects (two-factor) analysis of variance. Significance was set at *P* < 0.05.

## Results

A total of 52 anesthetic records of adult horses receiving anesthetized left-sided prosthetic laryngoplasty between June 2016 and March 2017 were evaluated for study inclusion. Eleven blocked horses were excluded from analysis on the basis of the C2–C3 block administered by alternate anesthetists (6/11), laryngoplasty performed by an alternate surgeon (1/11), incomplete data (2/11), a volume >10 mL 0.5% bupivacaine HCl administered (1/11), and administration of the C2–C3 block at the conclusion of surgery (1/11). A single non-blocked horse was excluded on the basis of incomplete data (1/1). The remaining horses for analysis included 20 horses receiving the C2–C3 block and 20 control (non-blocked) horses. All horses within the study were preoperatively classified as ASA physical status I. Of the 20 blocked horses, all were thoroughbred racehorses, aged of 2.8 ± 0.8 years (median, 3 years; range 2–4 years) and included 6 colts, 13 geldings, and 1 filly. No intraoperative/postoperative complications were noted secondary to the C2–C3 block. Of the 20 non-blocked horses, all were thoroughbred racehorses, of a mean age of 3.2 ± 1.1 years (median, 3 years; range, 2–6 years), and included 8 colts, 7 geldings, and 5 fillies. Of all horses anesthetized, 22/40 (55%) were maintained on halothane inhalant, 17/40 (43%) were maintained on isoflurane inhalant, and a single horse (2.5%) received a combination of halothane/isoflurane inhalant due to the development of a cardiac dysrhythmia midprocedure. This horse was switched from halothane to isoflurane inhalant. Of the 20 blocked horses, 9/20 (45%) received halothane inhalant; 10/20 (50%), isoflurane inhalant; and 1/20 (5%), a combination of halothane/isoflurane inhalants. Of the 20 non-blocked horses, 13/20 (65%) received halothane inhalant; and 7/20 (35%), isoflurane inhalant.

There was no significant difference in direct MAP or heart rate measured throughout surgical stimulation between groups (Table 1; *P* = 0.25 and *P* = 0.07, respectively). Equal numbers of horses (16/20, 80%) were administered a constant rate infusion of dobutamine for cardiovascular support (Table 2; *P* > 0.99). No significant difference in the proportion of horses that moved or exhibited nystagmus during surgical stimulation was found (Table 2; *P* > 0.99).

**Table 1:** Continuous data analysis of horses undergoing anesthetized left laryngoplasty comparing the administration of an ipsilateral C2–C3 locoregional anesthetic block vs. no locoregional anesthetic block.

**Anesthetic parameter**	**Blocked (*n* = 20^b^)**	**Non-blocked (*n* = 20)**	***P*-value**
Vaporizer setting, pre-surgical (halothane)	2.57 ± 0.11	2.55 ± 0.18	>0.999
Vaporizer setting, pre-surgical (isoflurane)	2.57 ± 0.10	2.52 ± 0.08	>0.999
Vaporizer setting, surgical (halothane)	2.21 ± 0.15	2.12 ± 0.16	>0.999
Vaporizer setting, surgical (isoflurane)	2.08 ± 0.11	2.18 ± 0.12	>0.999
Mean arterial pressure, pre-surgical	57 (48–68)	57 (51–72)	0.95
Mean arterial pressure, surgical	69 ± 6	72 ± 7	0.25
pVar^a^ mean arterial pressure, surgical	20 (2.2–167)	29 (2.1–159)	0.25
Heart rate, pre-surgical	34 (30–39)	33 (29–38)	0.12
Heart rate, surgical	32 ± 2	30 ± 4	0.07
pVar^a^ heart rate, surgical	1.4 (0.21–9.6)	2.3 (0.07–7.9)	0.49
Presurgical anesthetic time	47 ± 11	45 ± 9	0.51
Surgical anesthetic time	66 ± 10	64 ± 7	0.47
Total anesthetic time	113 ± 17	109 ± 11	0.36
Recovery time	59 ± 23	71 ± 35	0.23

a *pVar, population variance*.

b *A single horse was removed from vaporizer dial setting analysis due to an intraoperative switch from halothane to isoflurane anesthetic secondary to development of a cardiac dysrhythmia*.

**Table 2:** Categorical data analysis of horses undergoing anesthetized left laryngoplasty comparing the administration of an ipsilateral C2–C3 locoregional anesthetic block vs. no locoregional anesthetic block.

**Anesthetic parameter**	**Block (*n* = 20)**	**Non-blocked (*n* = 20)**	**Odds ratio (95% confidence interval)**	***P*-value**
Halothane anesthetic used	9/20 (45%)	13/20 (65%)		
Isoflurane anesthetic used	10/20 (50%)	7/20 (35%)		
Halothane/isoflurane anesthetic used	1/20 (5%)	0/20 (0%)		
Dobutamine administration	16/20 (80%)	16/20 (80%)		>0.99
Movement	2/20 (10%)	3/20 (15%)		>0.99
Nystagmus	3/20 (15%)	3/20 (15%)		>0.99
Movement or nystagmus	4/20 (20%)	5/20 (25%)		>0.99
1 adjunct anesthetic administration	7/20 (35%)	17/20 (85%)	11 (2.3–40)	0.03^a^
≥2 adjunct anesthetic administration	3/20 (15%)	7/20 (35%)		0.27
Increase inhalant adjunct	4/20 (20%)	8/20 (40%)		0.3
PIVA^b^ adjunct	4/20 (20%)	13/20 (65%)	7.4 (1.8–25)	0.01^a^
Intravenous anesthetic adjunct	3/20 (15%)	5/20 (25%)		0.69
Recovery sedation/analgesia	0/20 (0%)	16/20 (80%)		N/A
Recovery quality (good vs. other than good)	16/20 (80%)	15/20 (75%)		>0.99
Halothane	7/9 (80%)	10/13 (77%)		>0.99
Isoflurane	8/10 (80%)	5/7 (71%)		>0.99
Halothane/Isoflurane	1/1 (100%)	0/20 (0%)		N/A

a *Data are significantly different P < 0.05*.

b *PIVA, partial intravenous anesthesia*.

No significant difference was identified between the mean vaporizer dial setting (VDS) of inhalant anesthetic between either blocked horses and non-blocked horses during surgical stimulation comparing both halothane (Table 1; *P* > 0.99) and isoflurane (Table 1; *P* > 0.99). A single horse from the blocked group that received both halothane and isoflurane inhalant was excluded from VDS analysis. Non-blocked horses were 11 times more likely to require a defined adjunct anesthetic during surgical stimulation [Table 2; odds ratio (OR) = 11, 2.3–40; *P* = 0.03] and were 7.4 times more likely to require administration of partial intravenous anesthesia in addition to volatile anesthetic during surgical anesthesia (Table 2; OR= 7.4, 1.8–25; *P* = 0.01). No blocked horses received additional recovery sedation/analgesia as compared to 16/20 (80%) of non-blocked horses (Table 2).

The mean presurgical anesthetic time was slightly increased in blocked horses (47 ± 11 min) vs. non-blocked horses (45 ± 9 min), but the difference was not significant (Table 1; *P* = 0.51). The surgical anesthetic time was slightly increased in blocked horses (66 ± 10 min) vs. non-blocked horses (64 ± 7 min) m, but the difference was not significant (Table 1; *P* = 0.47). The overall total anesthetic time was not significant between groups (Table 1; *P* = 0.36).

No significant difference in recovery time was observed between blocked horses (60 ± 23 min) vs. non-blocked horses (71 ± 35 min), horses maintained under anesthesia using halothane (72 ± 31 min) vs. isoflurane (58 ± 28 min) inhalant, or when accounting for inhalant administered and whether a C2–C3 block was administered (*P* > 0.05). No significant difference in recovery quality between blocked vs. non-blocked horses was observed when recoveries were classified as “good” or “other than good” (Table 2; *P* > 0.99). No significant difference was observed between horses anesthetized with halothane (17/22, 77%) vs. isoflurane (14/17, 82%) when recoveries were classified as “good” or “other than good” (*P* = 0.46) regardless of C2–C2 block status. All horses recovered from general anesthesia with no reported complications.

## Discussion

Results of our study found that the use of a C2–C3 ipsilateral locoregional block was associated with a reduced intraoperative adjunctive anesthetic requirement. However, there was no significant difference in recovery quality noted between groups. We found a decreased incidence of additional sedation/analgesia administration prior to recovery in horses in which locoregional anesthesia was performed without reduction in quality of recovery. With a reported prevalence of anesthesia-related mortality ranging between 0.12 and 1%, any effort to improve general anesthesia quality should be maximized (12, 13). The clinical report here describes the use and potential benefit of ipsilateral cervical spinal nerve locoregional anesthesia in anesthetized horses undergoing prosthetic laryngoplasty to reduce anesthetic requirement and improve anesthetic quality.

Minimum alveolar concentration (MAC) is the volatile anesthetic concentration necessary to prevent purposeful movement in 50% of patients in response to a noxious stimulus (14, 15). Minimum alveolar concentrations are anesthetic agent dependent, but the proportion of MAC required for anesthesia can vary between patients, and thus, it is a guideline (15, 16). Increasing the alveolar concentration of a volatile anesthetic predisposes to dose-dependent side effects. “MAC sparing” strategies reduce the proportion of volatile anesthetic required to maintain surgical anesthesia and therefore dose-dependent side effects (3, 4, 8, 17–19). During general anesthesia monitoring, end-tidal volatile anesthetic concentration is a MAC proxy and a way to more accurately determine the amount of volatile anesthetic delivered. A limitation of this analysis is that the percent end-tidal volatile anesthetic was not routinely measured at the time under clinical practice conditions. Instead, the VDS was used as a crude estimate of total volatile anesthetic delivered and inference of alveolar concentration required to maintain surgical anesthesia in this clinical setting. As described, changes in the VDS and/or use of adjunctive anesthesia/analgesia were based on assessment of response to surgical stimulation. Results indicated no significant difference between groups in average surgical VDS; however, more non-blocked horses required additional anesthetic to maintain surgical anesthesia (both use adjunct anesthetic and PIVA; Table 2; *P* = 0.03 and *P* = 0.01, respectively). Prospective studies assessing the effect of C2–C3 on MAC reduction via end-tidal agent monitoring are needed to more accurately quantify a decrease in volatile anesthetic requirement.

We found no statistical difference in both presurgical and surgical MAP and heart rate between blocked and non-blocked horses. In fact, there was a trend for non-blocked horses to have lower heart rate during surgery compared to blocked horses (*P* = 0.07). While antinociception might theoretically reduce sympathoadrenal responses to surgical stimulation, the reasons for blocked horses to not have lower heart rates are not intuitive. Despite similar numbers of horses receiving operative dobutamine administration, (*P* > 0.99), total cumulative doses were not reported. One could speculate that blocked horses receiving predominantly volatile anesthetic alone may have required more dobutamine to maintain adequate MAP and cause small but present increases in heart rate. Per protocol at R.E.C., adjunct anesthesia is administered at the anesthetist's discretion based on assessment of anesthetic depth including elevations in MAP and heart rate to maintain appropriate surgical anesthesia. Additional anesthetic agents have profound effects on both MAP and heart rate, making direct comparisons between treated and untreated horses challenging. Nonetheless, a prospective controlled study evaluating the effect of infraorbital locoregional anesthesia on isoflurane MAC reduction in canines showed a significant reduction in MAC (*P* = 0.001) in subjects receiving locoregional anesthesia, yet no significant difference was observed in either MAP or heart rate between blocked and non-blocked subjects in response to noxious stimuli (8).

The risk avoidance of general anesthesia and recovery is cited as a major benefit in the advent of the standing prosthetic laryngoplasty (20). Indeed, standing surgery does not require anesthetic recovery, but standing laryngoplasty is performed under sedation using α2-adrenergic receptor agonists as either intermittent boluses or a constant rate infusion (11, 20). A recent study comparing fecal output and postoperative colic in horses undergoing standing or general anesthesia showed that horses with higher cumulative doses of detomidine had a longer time to first fecal passage and reduced overall 24-h fecal output. In that study, horses undergoing standing surgery uniformly received a higher total cumulative dose of detomidine, highlighting that standing surgery is not without risk of dose-dependent side effects (21). Our retrospective data support a reduced dependence on sedative analgesia or adjunctive anesthesia provision in horses receiving C2–C3 locoregional blockade, therefore potentially decreasing dose-dependent side effects of additional agents commonly used in anesthetic protocols. Again, a prospective randomized study is needed to confirm the reduced reliance on these medications and investigate the incidence of dose-dependent anesthetic-related morbidity.

Dugdale and colleagues' retrospective analysis of 1,416 equine anesthetic recoveries identified a significant improvement in recovery quality with administration of sedation in the early recovery period (13). A validated numerical scale based on descriptive terms was used in this determination. Historically, horses undergoing laryngoplasty at R.E.C. were thought to experience poorer recoveries comparatively because of an increased anesthetic requirement in response to surgical stimulus and/or increased postoperative pain. The basis was the observation of improved recovery quality with the administration of neuroleptanalgesia, whether it stemmed from the analgesic or sedative properties. Similarly, Clark et al. (22) found equine elective surgical cases anesthetized with halothane required fewer attempts and stood in a shorter period of time when administered intraoperative morphine. In this analysis, no horses administered locoregional anesthesia received additional sedative/analgesic drugs prior to recovery yet recovered with equal quality to non-blocked horses, of which the majority did (Table 2; 16/20, 75%). Suggesting nociception blocked through locoregional anesthesia may decrease the additional recovery sedation/analgesic requirement. At R.E.C., a non-validated trichotomous descriptive subjective scale was used to determine recovery quality. Using a non-validated recovery quality scale could have limited this conclusion because of increased subjectivity causing increased intraobserver and interobserver variability. However, the simplicity makes the intraobserver and interobserver agreement likely more robust. In one study, two descriptive objective scales were found to be in agreement with a descriptive subjective scale (23). Another identified reliability and reproducibility among multiple validated recovery quality scoring systems (24). Even more so, all scoring systems have limitations inferenced by the lack of a universally accepted validated scale (23).

Contrarily, inferior anesthetic recovery quality has been linked to increased duration of anesthesia (13). Performing the C2–C3 block did not significantly increase presurgical time in this patient population (Table 1; *P* = 0.51). By performing this after anesthetic induction and patient compliance to blockade coupled with skilled operators performing the block, there is little concern C2–C3 would unnecessarily increase total anesthesia time as seen in our population (Table 1; *P* = 0.36). Although not significant, non-blocked horses on average remained recumbent in recovery longer than blocked horses. This in part could be due to the higher proportion of non-blocked horses receiving halothane inhalant, which although non-significant tended to have a longer recovery time as compared to horses receiving isoflurane. Additionally, non-blocked horses were more likely to receive PIVA that can accumulate within the tissues and additional sedation/analgesia prior to recovery, both of which could prolong recumbency. Recovery scoring systems often classify increased duration of recovery as a marker for poorer recovery quality; however, this can be debated, and prospective studies evaluating the morbidity associated with recovery length are warranted (23).

No C2–C3 block-associated complications were reported in this analysis. The ventral branch of C2 and the cutaneous branches of C3 do not provide sensory innervation to the deeper laryngeal structures. During standing prosthetic laryngoplasty, a second intraoperative local anesthetic infiltration at the level of the caudal pharyngeal constrictors and cricoarytenoid joint is necessary for antinociception (11). Albeit rare, anecdotal transient contralateral arytenoid paresis/plegia has been observed during standing prosthetic laryngoplasty due to local anesthetic diffusion leading to inadvertent blockade of the contralateral recurrent laryngeal nerve. Whether it is diffusion of the secondary infiltration or the C2–C3 block resulting in contralateral recurrent laryngeal nerve blockade, altered cricoarytenoideus dorsalis function and transient contralateral arytenoid dysfunction have not been elucidated. In the conscious standing horse, intraoperative endoscopic monitoring easily identifies contralateral arytenoid dysfunction. To maintain adequate airflow through the rima glottidis, the ipsilateral arytenoid can be held intraoperatively in hyperabduction until transient dysfunction ceases. In the unconscious anesthetized horse, the presence of contralateral arytenoid dysfunction cannot be confirmed prior to recovery. Arytenoid collapse on extubation can cause significant airway obstruction, resulting in respiratory distress and death if a patent airway is not reestablished. Thus, secondary infiltration was not performed, and the injectate volume reduced to limit inadvertent diffusion to the contralateral side in this clinical report. While the axonal length of bathing necessary to achieve nociceptive blockade of C2 in horses has not been determined, blockade of C2 in humans using a blind technique is achieved with 3 to 5 mL of local anesthetic (25). Additionally, ultrasound-guided infiltration of 5 mL of new methylene blue in a separate fresh cadaver pilot study performed by author T.B.M. confirmed bathing of the major branches of C2. A limitation of this report as well as the original description is that the reliance of clinical response to surgical stimulation has been the main criterion used to assess efficacy.

The retrospective nature of this report presents limitations. Of those not previously addressed, the use of different volatile anesthetics is one. At the time the study was conducted, halothane inhalant was still available and used where the study was performed; however, the practice was transitioning to include the use isoflurane inhalant. Horses in both groups were anesthetized using either halothane or isoflurane inhalants with no difference between volatile anesthetic used observed including recovery quality and therefore were included in analysis. Variability among those performing the block, monitoring anesthesia, and performing surgery is another. Including only cases where both the C2–C3 block and the laryngoplasty procedure were performed by the same anesthetist or surgeon throughout decreased such variability. Unfortunately, it was not possible to control for the fact that multiple anesthetists were responsible for the intraoperative and recovery case management. Despite the instruction of and/or direct supervision by a diplomate of the American College of Veterinary Anesthesia and Analgesia (C.I.D.), the lack of a standardized approach to intervention triggers, anesthetic drug use, and recovery protocols could have introduced variability that confounds the findings of this study. Additionally, the retrospective study design without randomization of horses receiving C2–C3 block or no block introduces bias to the patient population and potentially clinical decision making of the anesthetist. Bias toward the overall type and quantity of anesthetic agent(s) used may have been introduced on the knowledge that a particular horse received additional local anesthetic.

Regardless of these limitations, we found that C2–C3 locoregional anesthesia was associated with a decreased need for additional intraoperative adjunct anesthetic agents, and there was no difference in recovery despite non-administration of sedation/analgesia prior to recovery. As such, it has been adopted into clinical practice at R.E.C. for horses undergoing prosthetic laryngoplasty. Future prospective randomized controlled studies investigating the effect of C2–C3 block on MAC reduction, total drug requirement, validated recovery scores, and postoperative pain assessment in horses undergoing anesthetized prosthetic laryngoplasty are required.

## Data Availability Statement

The dataset for this article is not publicly available because data was obtained from confidential patient medical records during retrospective analysis. Requests to access the datasets should be directed to Tate B. Morris, tbmorris@vet.upenn.edu.

## Ethics Statement

Ethical review and approval was not required for the animal study as it was a retrospective analysis of clinical data obtained from medical records of client owned animals. Owner consent was obtained prior to surgical procedures and standard of care upheld. The local anesthetic technique evaluated has been established in a peer-reviewed journal.

## Author Contributions

CD, JL, and TM contributed conception and design of the study. SS performed locoregional anesthetic blocks on clinical cases. JL performed prosthetic laryngoplasty on clinical cases. VL provided medical records, anesthetic protocol, and product information. TM organized the database and wrote the first draft of the manuscript. SH performed the statistical analysis and wrote the statistical analysis section of the manuscript. All authors contributed to manuscript revision, read, and approved the submitted version.

## Conflict of Interest

SH and TM are affiliated with the same institution as Recent Advancement in Equine Anesthesia topic editors Bernd Driessen and Klaus Hopster: School of Veterinary Medicine, University of Pennsylvania, Philadelphia, United States. The remaining authors declare that the research was conducted in the absence of any commercial or financial relationships that could be construed as a potential conflict of interest.
